# Двухэнергетическая компьютерная томография в задаче дифференциальной диагностики образований надпочечников

**DOI:** 10.14341/probl13671

**Published:** 2025-12-02

**Authors:** Н. В. Тарбаева, А. В. Манаев, А. Ю. Корнелюк, М. В. Годзенко, К. В. Иващенко, А. Шевэ, Г. А. Мельниченко, Н. Г. Мокрышева

**Affiliations:** Национальный медицинский исследовательский центр эндокринологии им. академика И.И. ДедоваРоссия; Endocrinology Research CentreRussian Federation

**Keywords:** двухэнергетическая компьютерная томография, классификация, образование надпочечника, адренокортикальный рак, аденома, феохромоцитома, dual-energy computed tomography, classification, adrenal lesion, adrenocortical carcinoma, adenoma, pheochromocytoma

## Abstract

**ОБОСНОВАНИЕ:**

ОБОСНОВАНИЕ. Дифференциальная диагностика образований надпочечников (аденом, адренокортикального рака (АКР), феохромоцитом) остается сложной задачей из-за схожести их характеристик при стандартной компьютерной томографии (КТ). Двухэнергетическая КТ (ДЭКТ) обладает потенциалом для улучшения дифференциации за счет анализа спектральных характеристик, однако ее роль в диагностике образований надпочечников изучена не в полной мере.

**ЦЕЛЬ:**

ЦЕЛЬ. Исследование диагностической ценности характеристик ДЭКТ в дифференциальной диагностике образований надпочечников.

**МАТЕРИАЛЫ И МЕТОДЫ:**

МАТЕРИАЛЫ И МЕТОДЫ. Проведено одноцентровое ретроспективное исследование (данные ДЭКТ за 2023–2024 гг.). Анализировали параметры монохроматических изображений 40, 70 и 80 кэВ, карт разложения вода-йод, жир-йод и вода-жир. Статистический анализ включал тесты Краскела-Уоллиса, Манна-Уитни, ROC-анализ.

**РЕЗУЛЬТАТЫ:**

РЕЗУЛЬТАТЫ. Выборка включала 74 пациента с данными ДЭКТ (медианный возраст — 46,0 лет, межквартильный интервал (39,3–57,9); 48 женщин). Наибольшая дискриминативная способность для дифференциации АКР и аденом высокой плотности выявлена у среднего значения рентгеновской плотности изображений 70 кэВ в отсроченной фазе (AUC=0,92; 95% доверительный интервал (ДИ): 0,82–0,99; p=0,001), а для АКР и феохромоцитом — у стандартного отклонения концентрации воды на карте вода-жир в отсроченной фазе (AUC=0,89; 95% ДИ: 0,79–0,97; p=0,001). Для дифференциации аденом и феохромоцитом ключевым признаком стал максимум рентгеновской плотности на изображении 70 кэВ в отсроченной фазе (AUC=0,82; 95% ДИ: 0,72-0,92; p<0,001).

**ЗАКЛЮЧЕНИЕ:**

ЗАКЛЮЧЕНИЕ. ДЭКТ демонстрирует высокую диагностическую точность в дифференциации образований надпочечников.

## Abstract (en)

**BACKGROUND:**

BACKGROUND: Adrenal lesion characterization remains a diagnostic challenge due to overlapping imaging features. Dual-energy CT (DECT) shows promise for identifying image-based biomarkers but requires further validation.

**AIM:**

AIM: To evaluate the diagnostic utility of DECT in differentiating adrenal lesions.

**MATERIALS AND METHODS:**

MATERIALS AND METHODS: This retrospective single-center study analyzed DECT data. Parameters from 40/70/80 keV virtual monoenergetic images and water-iodine/fat-iodine/water-fat decomposition maps were evaluated. Statistical analysis included Kruskal-Wallis, Mann-Whitney U, ROC tests.

**RESULTS:**

RESULTS: The cohort included 74 patients (median age 46.0 years (39.3–57.9), 48 women). For ACC and adenomas differentiation, mean 70 keV delayed phase attenuation showed the highest discriminative value (AUC=0.92; 95% CI: 0.81–1.00; p=0.001). For ACC and pheochromocytomas, standard deviation delayed phase water concentration (water-fat maps) achieved the highest diagnostic performance (AUC=0.89; 95% CI: 0.79–0.97; p=0.001). Maximum 70 keV delayed phase attenuation best distinguished adenomas from pheochromocytomas (AUC=0.82; 95% CI: 0.71–0.92; p<0.001).

**CONCLUSION:**

CONCLUSION: DECT has proven its diagnostic value in the differential diagnosis of adrenal lesions.

## ОБОСНОВАНИЕ

Дифференцирование инциденталом надпочечников, выявляемых различными методами инструментальной диагностики, представляет собой диагностическую проблему, имеющую важное значение для определения дальнейшей тактики лечения и прогноза пациента. По данным литературы, при компьютерной томографии (КТ) частота обнаружения инциденталом достигает 3–8% [1]. Хотя КТ с контрастным усилением (КУ) считается «золотым стандартом» для визуализации опухолей надпочечников, ее эффективность ограничена при дифференцировании аденом и злокачественных новообразований — адренокортикального рака (АКР) и феохромоцитом из-за значительного совпадения их характеристик на КТ [2]. В свою очередь, двухэнергетическая КТ (ДЭКТ) или спектральная КТ — это модификация КТ-исследования, при котором сканирование осуществляется при двух различных уровнях энергии рентгеновского излучения. Это позволяет выходить за рамки оценки рентгеновской плотности и дает возможность различать материалы за счет различного коэффициента ослабления рентгеновского излучения при разных энергиях разными тканями. ДЭКТ позволяет реконструировать виртуальные неконтрастные изображения (ВНИ), монохроматические изображения и карты распределения концентраций различных материалов (например, пары жир-вода, жир-йод, вода-йод) [3].

В зарубежной литературе имеются единичные, представляющие научный интерес публикации, посвященные применимости ДЭКТ в дифференциальной диагностике образований надпочечников, преимущественно — дифференциации аденом от метастазов. Так, в исследовании [4] проведен анализ изображений венозной фазы 160 образований надпочечников размером более 10 мм (104 аденомы, 56 метастазов). Получено, что значение концентрации йода аналогично рентгеновской плотности нативных изображений КТ с точки зрения дифференцирования аденом и метастазов (чувствительность 78%, специфичность 71%), в то время, как характеристика «концентрация йода»/«рентгеновская плотность ВНИ» продемонстрировала лучшую дискриминативную способность с чувствительностью 95% и специфичностью 95% при использовании порога 6,7. Кроме того, в исследовании по изучению возможностей ДЭКТ в дифференциальной диагностике аденом надпочечников от неаденом (преимущественно метастазы) [5] было показано, что значение жировой фракции, вычисляемое на основе алгоритма разложения на 3 компонента в ДЭКТ (вода, йод и жир) в венозной фазе КТ, обладает более высокой чувствительностью в выявлении аденом по сравнению с традиционными пороговыми значениями рентгеновской плотности на нативных изображениях. Так, при 100% специфичности чувствительность выявления аденом по жировой фракции составила 59%, тогда как при использовании порога ≤10 HU на нативных изображениях — 28%.

Также имеются публикации по исследованию применимости ДЭКТ в дифференциации аденом от феохромоцитом. В публикации [6] анализировались изображения ДЭКТ пациентов с патологически подтвержденными аденомами надпочечников (70 случаев) и феохромоцитомами (15 случаев). Авторы реконструировали виртуальные моноэнергетические изображения (ВМИ) при 40, 70 и 100 кэВ и исследовали различные параметры, включая концентрацию йода в артериальной и венозной фазах КТ, эффективное атомное число и наклон графика зависимости рентгеновской плотности в единицах Хаунсфилда (HU) от величины энергии. ROC-анализ продемонстрировал высокую диагностическую точность ВМИ при энергии 40 кэВ, концентрации йода в артериальной фазе и наклон графика зависимости рентгеновской плотности в HU от величины энергии. Значения AUC составили 0,818, 0,736 и 0,817 соответственно для дифференцировки аденом от феохромоцитом.

Важно отметить, хотя ДЭКТ и продемонстрировала перспективность в дифференциации различных поражений надпочечников на примере аденом и метастазов, аденом и феохромоцитом, в существующих работах нет конкретных доказательств ее применимости для диагностики других классов образований надпочечников, таких, как АКР. В свою очередь, уже накопленный опыт применения ДЭКТ доказывает, что методика может привести к сокращению количества исследований, снижению лучевой нагрузки, общей экономии средств и улучшению качества диагностики пациентов, что указывает на необходимость дальнейших исследований.

## ЦЕЛЬ ИССЛЕДОВАНИЯ

Исследование диагностической ценности характеристик ДЭКТ в дифференциальной диагностике образований надпочечников.

## МАТЕРИАЛЫ И МЕТОДЫ

## Место и время проведения исследования

Место проведения. ФГБУ «НМИЦ эндокринологии им. академика И.И. Дедова» Минздрава России, референс-центр лучевых методов диагностики.

Время исследования. С ноября 2024-го по август 2025 гг.

## Изучаемые популяции (одна или несколько)

Целевая популяция определялась критериями включения и исключения.

Критерии включения: наличие в медицинской базе данных результатов проведения ДЭКТ в ФГБУ «НМИЦ эндокринологии» Минздрава России (далее — Центр) в нативную, артериальную, венозную и отсроченную фазу КУ; возраст≥18 лет; наличие патоморфологического заключения.

Критерии исключения: наличие артефактов движения на изображениях ДЭКТ в области надпочечников; диагноз, отличный от аденомы, феохромоцитомы, адренокортикальный рак; максимальная плотность солидного компонента менее 20 HU в случае аденом.

Блок-схема формирования выборки исследования приведена на рисунке 1.

**Figure:**
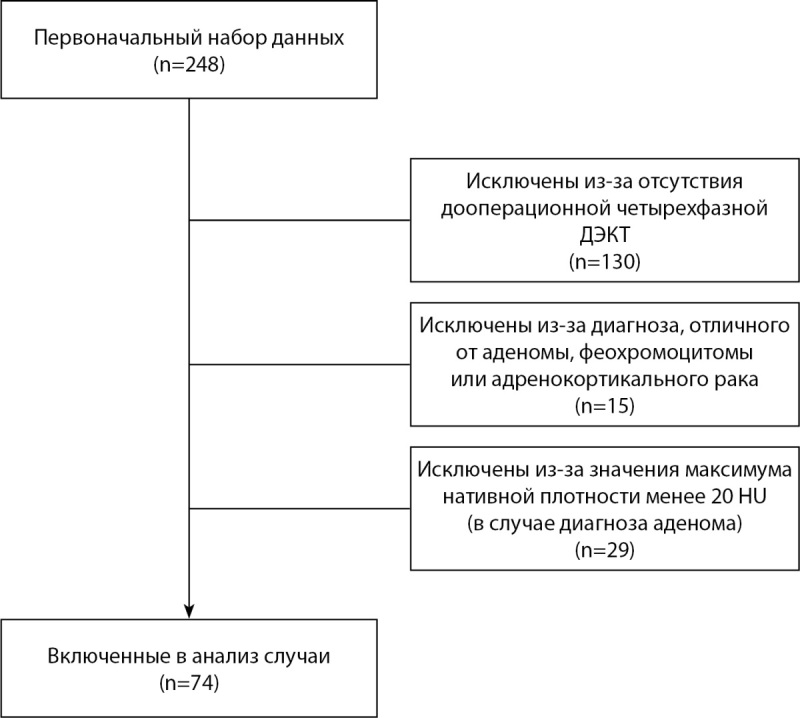
Рисунок 1. Блок-схема формирования выборки.

## Способ формирования выборки из изучаемой популяции (или нескольких выборок из нескольких изучаемых популяций)

Участники исследования подбирались в соответствии с критериями отбора до включения в исследование с формированием «удобной» для наблюдения исследователями выборки пациентов с образованиями надпочечников, зарегистрированных в медицинской информационной системе.

## Дизайн исследования

Одноцентровое несравнительное одномоментное ретроспективное исследование.

## Получение КТ-изображений и извлечение характеристик ДЭКТ

Изображения получены при положении пациента на спине с использованием компьютерного томографа Revolution CT (GE Healthcare, США) с использованием метода быстрого чередования напряжения на рентгеновской трубке между двумя уровнями 80 и 140 кВ во время одного оборота трубки. Сила тока на трубке 485 мА в артериальной и венозной фазе, 280 мА — в отсроченной фазе, 200 мА — в нативной фазе КТ. Сканирование выполнялось в спиральном режиме. Ширина коллимации — 128x0,625 мм, толщина среза — 0,625/1,25 мм, интервал между срезами — 1,25 мм, питч — 0,992.

Контрастное усиление осуществлялось с использованием двухколбового автоматического инжектора Medrad Stellant (Bayer, Германия) со скоростью подачи 3,5–4 мл/с в течение всего периода сканирования. Артериальная фаза проводилась через 10 секунд после срабатывания болюсного триггера, установленного на нисходящей части аорты на уровне диафрагмы (120 HU). Венозная фаза выполнялась на 30-й секунде от момента триггера, а отсроченная — через 10–15 минут после введения контрастного вещества.

Данные ДЭКТ получены с использованием специализированного программного обеспечения AW Server 3.2 Ext 6.0 (GE Healthcare, США). Оценка рентгеновской плотности и характеристик тканевых структур осуществлялась по монохроматическим изображениям 40, 70, 80 кэВ, картам двухкомпонентного разложения йод-вода, йод-жир и вода-жир.

Определение характеристик ДЭКТ проводил врач-рентгенолог с опытом работы более 5 лет. Проводился слепой анализ без доступа к клинической информации во избежание объект-ориентированных различий в интерпретации изображений ДЭКТ. Среднее, максимальное и минимальное значение рентгеновской плотности на изображениях 40, 70 и 80 кэВ, концентрации всех материалов в картах вода-йод, жир-йод и вода-жир измерялись путем ручного размещения округлой области интереса. Область интереса размещалась внутри образований надпочечников, охватывая максимально возможную сплошную зону на уровне сечения с максимальным диаметром очага, при этом избегая некротических и кистозных участков, кальцификации, нормальной паренхимы надпочечников, окружающей жировой ткани и сосудов. Также проводили измерения аналогичной методикой тех же показателей ДЭКТ для здоровой ткани надпочечников. Анализу подверглись артериальная, венозная и отсроченная фазы ДЭКТ в рамках стандартного протокола Центра.

## Статистический анализ

Статистический анализ проводили с помощью языка программирования Python 3.9.21. В рамках описательной статистики количественные переменные выражены через медиану и межквартильный интервал, категориальные переменные выражены через частоту. Для сравнения количественных переменных среди анализируемых групп использовали тест Краскела-Уоллиса, для попарных сравнений применяли непараметрический тест Манна-Уитни, для сравнения категориальных переменных в группах — критерий χ².

Однофакторный анализ данных

Однофакторный анализ проводили в 2 этапа: сначала среди всех характеристик изображений и карт ДЭКТ с применением теста Краскела-Уоллиса выбрали те, которые характеризуются статистически значимыми различиями среди 3 классов: АКР, аденомы и феохромоцитомы. После отсечения неинформативных признаков проводили попарные сравнения с применением теста Манна-Уитни. На обоих этапах анализа проводили коррекцию на множественные сравнения. На первом этапе проводили глобальную коррекцию для всех тестов Краскела-Уоллиса с применением метода Бенджамини-Хохберга со значением FDR=0,05. На втором этапе проводили локальную коррекцию на множественные сравнения с применением поправки Бонферрони. В рамках однофакторного анализа данных для определения размера эффекта значимых признаков использовали метрики AUC, чувствительность и специфичность при попарных сравнениях, соответствующие максимальному значению индекса Юдена. Уровень значимости во всех случаях приняли равным 0,05.

Оценка 95% доверительного интервала

95% доверительные интервалы (95% ДИ) для значений медианы, чувствительности, специфичности и AUC в рамках однофакторного анализа оценивались с использованием непараметрического бутстрапа с количеством бутстрап-выборок, равным 1000.

## Этическая экспертиза

Исследование рассмотрено и одобрено Локальным этическим комитетом ФГБУ «НМИЦ эндокринологии им. академика И.И. Дедова» Минздрава России (код протокола 20, 13.11.2024). Все пациенты дали письменное согласие на использование результатов обследования и лечения с научной целью.

## РЕЗУЛЬТАТЫ

## Описание выборки

Среди 74 пациентов, включенных в исследование, у 48 пациентов (65%) было выявлено образование надпочечника справа, а у 26 (35%) — слева. Образования были разделены на 3 класса — аденомы высокой плотности (26 случаев), АКР (10 случаев) и феохромоцитомы (38 случаев). При анализе связи пола и возраста пациентов с классами образований надпочечников статистически значимой связи не обнаружено (табл. 1). В то же время имеется статистически значимая связь между объемом и классами образований надпочечников, наибольший объем характерен для АКР, наименьший — для аденом.

**Table:** Таблица 1. Описательная статистика выборки и результаты теста Краскела-Уоллиса/теста хи-квадрат

Характеристика	Диагноз	p-value
АКР	Аденома высокой плотности	Феохромоцитома
Медиана возраста (межквартильный интервал), лет	40,0 (33,4–42,5)	49,5 (40,8–61,1)	48,0 (40,0–57,4)	0,24
46,0 (39,3–57,9)
Медиана объема (межквартильный интервал), мл	128,0 (64,7–187,5)	11,0 (7,7–29,7)	36,8 (14,6–86,2)	<0,001
29,8 (10,7–86,2)
Количество женщин	7	15	26	0,63
Общее количество случаев	10	26	38

## Основные результаты исследования

Однофакторный анализ характеристик ДЭКТ

Анализировались данные 3 классов образований надпочечников (АКР, аденомы, феохромоцитомы).

После проведения тестов Краскела-Уоллиса и тестов Манна-Уитни с коррекцией на множественные сравнения согласно алгоритму, описанному в разделе «Однофакторный анализ данных», в таблице 2 приведены 5 наиболее информативных (по значению AUC) признаков для каждой пары анализируемых диагнозов.

**Table:** Таблица 2. Диагностическая ценность характеристик ДЭКТ Примечание: в таблицах для обозначений признаков приняты следующие сокращения: max — максимальное значение, min — минимальное значение, усреднение — среднее значение. При анализе карт концентраций веществ на первом месте в паре указан материал, для которого определяется признак.

Характеристика ДЭКТ	AUC (95% ДИ)	Чувствительность (95% ДИ)	Специфичность (95% ДИ)	Пороговое значение, для АКР/феохромоцитомы значение больше или меньше	p-value
Дифференциация аденом и АКР
Артериальная фаза
усреднение (жир (йод))	0,92 (0,81–1,00)	0,88 (0,76–1,00)	0,92 (0,65–1,00)	1021,00, >	0,001
max (вода (йод))	0,91 (0,79–1,00)	0,88 (0,75–1,00)	0,92 (0,62–1,00)	1054,00, >	0,002
Венозная фаза
усреднение (вода (йод))	0,91 (0,80–0,99)	1,00 (0,75–1,00)	0,69 (0,58–1,00)	1025,00, >	0,002
Отсроченная фаза
усреднение (изобр. 80 кэВ)	0,92 (0,82-0,99)	1,00 (0,88-1,00)	0,81 (0,69-0,96)	28,40, >	0,001
max (изобр. 80 кэВ)	0,91 (0,81–0,99)	1,00 (0,75–1,00)	0,69 (0,58–1,00)	60,00, >	0,002
Дифференциация феохромоцитом и АКР
Венозная фаза
усреднение (вода (йод))	0,89 (0,75–0,99)	0,75 (0,63–1,00)	0,90 (0,53–1,00)	1032,00, >	0,001
усреднение (жир (йод))	0,87 (0,74–0,97)	0,75 (0,623–1,00)	0,90 (0,47–0,97)	1023,00, >	0,001
Отсроченная фаза
стандарт откл. (вода (жир))	0,89 (0,79–0,97)	1,00 (0,88–1,00)	0,74 (0,63–0,95)	430,30, ≤	<0,001
стандарт откл. (йод (вода))	0,89 (0,78–0,97)	1,00 (0,88–1,00)	0,74 (0,63–0,95)	5,40, ≤	0,001
стандарт откл. (жир (вода))	0,88 (0,76–0,97)	1,00 (0,88–1,00)	0,74 (0,63–0,95)	418,70, ≤	0,001
Дифференциация феохромоцитом и аденом
Артериальная фаза
max (изобр. 70 кэВ)	0,82 (0,71–0,92)	0,82 (0,61–1,00)	0,77 (0,50–0,96)	108,00, >	<0,001
max (изобр. 80 кэВ)	0,82 (0,71–0,92)	0,79 (0,61–1,00)	0,73 (0,46–0,96)	61,00, >	<0,001
Отсроченная фаза
max (изобр. 70 кэВ)	0,82 (0,72–0,92)	0,84 (0,74–0,97)	0,77 (0,58–0,92)	110,00, >	<0,001
усреднение (изобр. 70 кэВ)	0,80 (0,69–0,91)	0,79 (0,55–0,97)	0,73 (0,54–0,96)	54,00, >	<0,001
усреднение (изобр. 80 кэВ)	0,79 (0,68–0,90)	0,71 (0,61–1,00)	0,81 (0,46–0,92)	28,00, >	<0,001

Наиболее информативными признаками для дифференциации АКР и аденом высокой плотности оказались характеристики с карт жир-йод (среднее значение концентрации жира) и вода-йод (среднее и максимальное значение концентрации воды) в артериальной и венозной фазе КТ. В отсроченной фазе наиболее информативными являются признаки карт 80 кэВ (среднее и максимальное значение рентгеновской плотности). При этом характеристикой с наибольшим значением AUC и при этом с максимальным значением чувствительности к АКР является среднее значение рентгеновской плотности на карте 80 кэВ в отсроченной фазе (AUC=0,92; 95% доверительный интервал (ДИ): 0,82–0,99; p<0,001) с пороговым значением 28,4 HU, что позволяет утверждать, что среднее значение рентгеновской плотности на карте 80 кэВ в отсроченной фазе более 28,4 HU характерно для АКР, а меньше или равно — для аденом высокой плотности (чувствительность 100% (95% ДИ 88–100%) и специфичность 81% (95% ДИ 69–96%)) (рис. 2).

**Figure:**
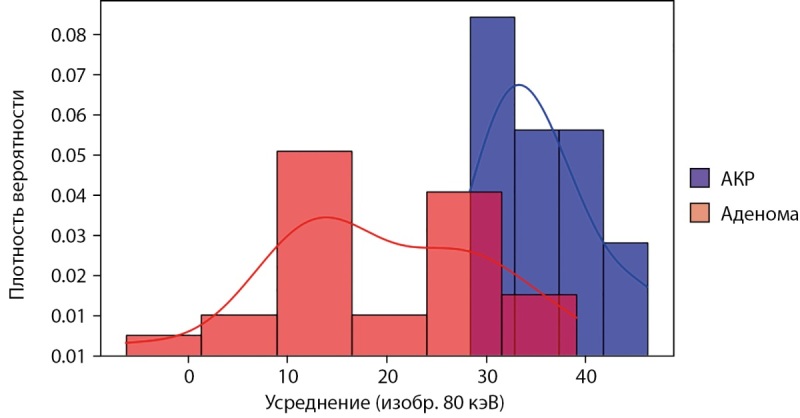
Рисунок 2. Гистограмма для среднего значения рентгеновской плотности на карте 80 кэВ в отсроченной фазе КТ.

В рамках задачи дифференциальной диагностики АКР и феохромоцитом наиболее информативными являются признаки с карт вода-йод (среднее значение концентрации воды) и жир-йод (среднее значение концентрации жира) в венозной фазе КТ и характеристики с карт вода-жир (стандартное отклонение концентрации воды), йод-вода (стандартное отклонение концентрации йода), жир-вода (стандартное отклонение концентрации жира) в отсроченной фазе. Признаком с максимальным значением AUC и наибольшей чувствительностью к АКР является стандартное отклонение концентрации воды на карте вода-жир в отсроченной фазе (AUC=0,89; 95% ДИ: 0,79–0,97; p=0,001) с пороговым значением 430,3 мг/см³, что позволяет утверждать, что стандартное отклонение концентрации воды на карте вода-жир в отсроченной фазе более 430,3 мг/см³ характерно для феохромоцитом, а меньше или равно — для АКР (чувствительность 100% (95% ДИ 88–100%) и специфичность 74% (95% ДИ 63–95%)) (рис. 3).

**Figure:**
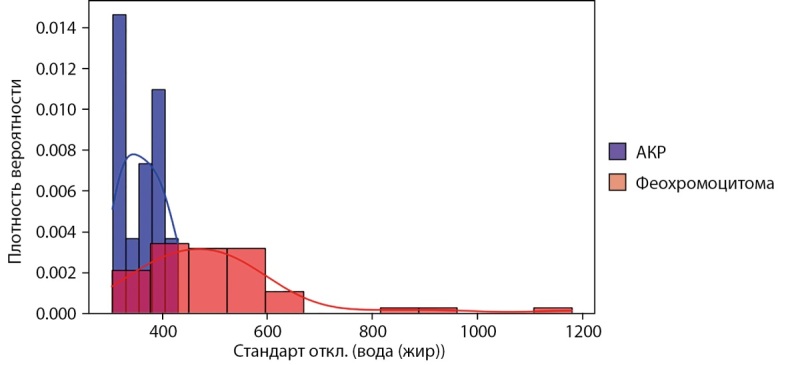
Рисунок 3. Гистограмма для стандартного отклонения концентрации воды на карте вода-жир в отсроченной фазе КТ.

Лучше всего для дифференциации феохромоцитом и аденом в анализируемой выборке показали себя признаки с карт 70 и 80 кэВ в артериальной и отсроченной фазах (максимальное и среднее значение рентгеновской плотности). Признаком с наибольшими значениями AUC и чувствительностью (к феохромоцитомам) является максимальное значение рентгеновской плотности на карте 70 кэВ в отсроченной фазе (AUC=0,82; 95% ДИ: 0,72–0,92; p<0,001) с пороговым значением 110 HU, что позволяет утверждать, что максимум рентгеновской плотности на карте 70 кэВ в отсроченной фазе более 110 HU характерен для феохромоцитомы, а меньше или равно — для аденомы высокой плотности (чувствительность 84% (95% ДИ 74–97%) и специфичность 77% (95% ДИ 58–92%)) (рис. 4).

**Figure:**
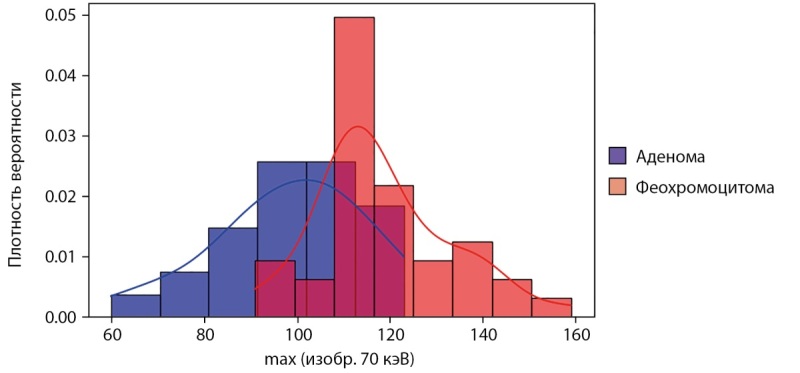
Рисунок 4. Гистограмма для максимального значения рентгеновской плотности на карте 70 кэВ в отсроченной фазе КТ.

## ОБСУЖДЕНИЕ

Согласно накопленному опыту, известно, что ДЭКТ является перспективным инструментом для описания образований надпочечников, однако на настоящее время ее диагностическая эффективность до конца не выяснена по причине немногочисленности выборки пациентов с результатами ДЭКТ в публикациях и трудозатратности процедуры сбора данных ДЭКТ. В настоящее время в зарубежной и отечественной литературе отсутствуют исследования по применимости ДЭКТ в дифференциальной диагностике адренокортикального рака (АКР) — одной из важнейших проблем эндокринологии.

Наши результаты показывают, что наиболее информативным параметром в дифференциальной диагностике АКР и аденом высокой плотности, является максимальное значение рентгеновской плотности солидного компонента опухоли на монохроматическом изображении 70 кэВ в отсроченной фазе. Анализируемая выборка характеризуется более высокими значениями данного признака для АКР по сравнению с аденомами (для АКР характерны значения более 28,4 HU). Полученный результат согласуется с ожиданиями и связан с тем, что АКР характеризуются замедленным вымыванием контрастного вещества, что проявляется более высокими значениями рентгеновской плотности в отсроченной фазе на карте 70 кэВ.

Также значимыми диагностическими критериями для дифференциальной диагностики аденом и АКР являются признаки с карт вода-йод и жир-йод. Примечательно, что анализируемая выборка характеризуется более высокими значениями концентрации жирового компонента в АКР по сравнению с аденомами (для АКР характерно среднее значение концентрации жира на карте жир-йод в артериальной фазе больше 1021 мг/см³). Ожидалось, что аденомы надпочечников содержат большее количество внутриклеточного жира, а АКР должен характеризоваться меньшим содержанием жира или его отсутствием — данный параметр используется для их идентификации на нативных изображениях КТ. Однако важно понимать, что результаты исследования, демонстрирующие более высокие значения концентрации жира в АКР по сравнению с аденомами надпочечников при двухкомпонентном разложении жир-йод, не отражают реальное содержание жирового компонента в тканях, а связаны с особенностями двухкомпонентного разложения в ДЭКТ и особенностями анатомии исследуемых тканей. Карта жир-йод предполагает, что каждый воксель содержит только два материала. Однако АКР также включает компоненты, неучтенные в модели: кровь, плотный солидный компонент, которые в силу ограничений двухкомпонентной модели интерпретируются как жир. Поэтому необходимо подчеркнуть, что значения на картах ДЭКТ могут использоваться с целью дифференциации образований надпочечников как количественный параметр, без привязки к реальной концентрации жира, в связи с несоответствиями предположений модели реальному составу образования. Кроме того, наш результат согласуется с результатами другой работы [7], в которой также получено, что аденомы (в том числе с высоким содержанием жира) характеризуются меньшим значением концентрации жира на картах жир-йод по сравнению с отличными от аденом образованиями надпочечников (в исследовании рассматривали метастазы).

Публикации по дифференциальной диагностике феохромоцитом и АКР также не представлены в зарубежной и отечественной литературе. В нашей работе показано, что наиболее информативным является стандартное отклонение концентрации воды в тканях опухоли на карте вода-жир в отсроченной фазе со значением AUC 0,89 (для феохромоцитом характерны значения больше 430,3 мг/см³). Полученный результат может быть связан с большей гетерогенностью структуры феохромоцитом с чередованием гиперваскулярных и гиповаскулярных зон.

В дифференциальной диагностике аденом и феохромоцитом с помощью ДЭКТ наиболее информативным признаком является максимальное значение рентгеновской плотности солидного компонента опухоли на изображении 70 кэВ в отсроченной фазе КТ со значением AUC 0,82 (для феохромоцитом характерны значения более 110 HU). Информативность карты 70 кэВ в отсроченную фазу в дифференциальной диагностике аденом и феохромоцитом, вероятно, связана со значительным накоплением контрастного вещества феохромоцитомами и возросшей вследствие этого ролью признаков, подчеркивающих эту разницу в накоплении контраста. Меньшая информативность карт концентраций различных пар материалов, хотя они, казалось бы, тоже должны подчеркивать разницу в накоплении йода, вероятно, связана с ограничениями двухкомпонентной модели разложений, которые уже обсуждались. В работе [6] по исследованию признаков ДЭКТ в дифференциальной диагностике аденом и феохромоцитом подчеркивается более высокая концентрация йода в феохромоцитомах в артериальную фазу. Наибольшей значимостью в данной работе характеризуются виртуальные монохроматические изображения 40 кэВ со значением AUC 0,818 (ДИ: 0,714–0,922). Кроме того, в данном исследовании подчеркивается, что низкий уровень энергии 40 кэВ обеспечивает лучшую дифференциацию аденом надпочечников и феохромоцитом по сравнению с более высокими энергиями 70 кэВ и 100 кэВ. В нашем исследовании информативность монохроматических изображений 70 и 80 кэВ оказалась выше, чем изображений 40 кэВ. Возможной причиной такого результата является факт того, что подходы к реконструкции монохроматических изображений в томографе Revolution CT производителя General Electric (который использовался в нашей работе) и для томографа IQon Spectral CT производителя Phillips (который использовался в работе [6]) значительно отличаются: если в General Electric используется метод быстрого переключения напряжения на рентгеновской трубке, что ведет к генерации рентгеновских спектров с различными энергетическими характеристиками, то в Phillips принцип реконструкции основан на использовании детекторов с энергетическим разрешением с регистрацией фотонов разных энергий без переключения напряжения на трубке. Имеется исследование [8], показывающее различия в реконструируемых изображениях различными методами спектральной компьютерной томографии, что доказывает правомерность вышеприведенных суждений.

Несмотря на статистическую значимость полученных результатов, исследования характеризуется рядом ограничений, которые можно структурировать следующим образом.

## ЗАКЛЮЧЕНИЕ

ДЭКТ доказала свою высокую диагностическую ценность в дифференциальной диагностике образований надпочечников. В сложных случаях, при наличии сомнительных результатов КТ, ДЭКТ позволяет получить дополнительную информацию, повышающую диагностическую точность метода в дифференциальной диагностике аденом, АКР и феохромоцитом.

## ДОПОЛНИТЕЛЬНАЯ ИНФОРМАЦИЯ

Источники финансирования. Работа выполнена по инициативе авторов без привлечения финансирования.

Конфликт интересов. Авторы декларируют отсутствие явных и потенциальных конфликтов интересов, связанных с содержанием настоящей статьи.

Участие авторов. Все авторы одобрили финальную версию статьи перед публикацией, выразили согласие нести ответственность за все аспекты работы, подразумевающую надлежащее изучение и решение вопросов, связанных с точностью или добросовестностью любой части работы.
